# Henle’s Ligament: A Comprehensive Review of Its Anatomy and Terminology over Almost One and a Half Centuries

**DOI:** 10.7759/cureus.3366

**Published:** 2018-09-26

**Authors:** Raja Gnanadev, Joe Iwanaga, Rod J Oskouian, Marios Loukas, R. Shane Tubbs

**Affiliations:** 1 Miscellaneous, Seattle Science Foundation, Seattle, USA; 2 Medical Education and Simulation, Seattle Science Foundation, Seattle, USA; 3 Neurosurgery, Swedish Neuroscience Institute, Seattle, USA; 4 Anatomy, St. George's University, St. George's, GRD; 5 Neurosurgery, Seattle Science Foundation, Seattle, USA

**Keywords:** friedrich henle, inguinal, henle’s ligament, conjoint tendon, conjoint area, falx inguinalis, anatomy

## Abstract

Henle’s ligament was first described by German physician and anatomist, Friedrich Henle, in 1871. This review article will cover Henle’s original description of the ligament, historical changes in terminology, embryological studies of the ligament, and the clinical significance of Henle’s ligament. This article has a particular focus on the variation in the terminology of this structure and the implications of this.

## Introduction and background

**Figure 1:**
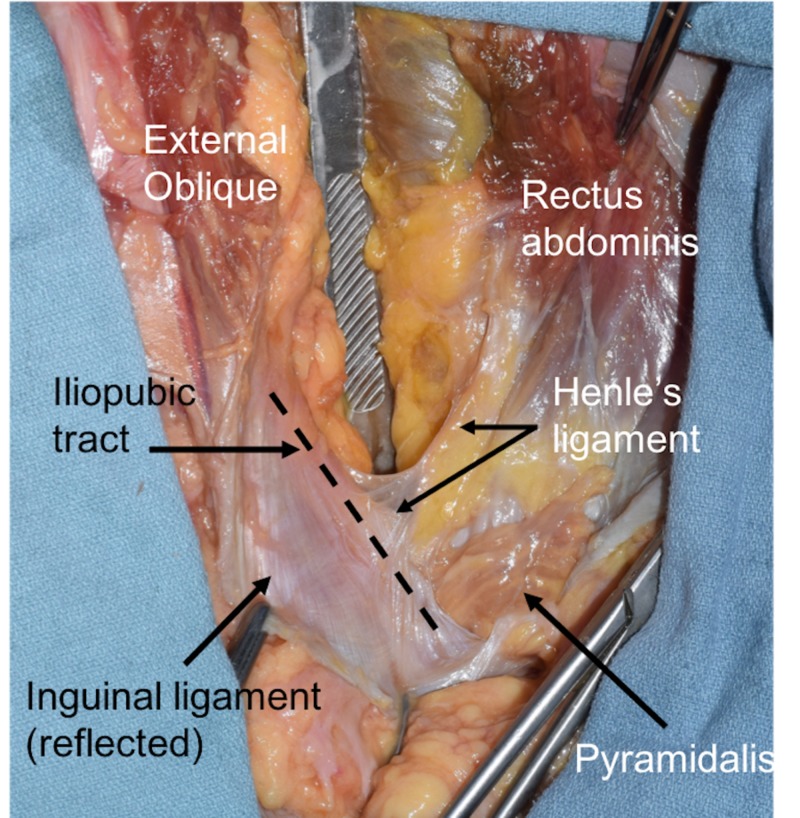
Right inguinal region dissected in a fresh frozen adult cadaver The scalpel handle is pushing down the contents of the peritoneal cavity and the inguinal ligament is cut and reflected inferiorly. The iliopubic tract is seen at the dotted line. An extension of the lateral aspect of the rectus abdominis muscle is seen as Henle’s ligament.

Scientific advancements of 19th century Europe were driven by the philosophies developed during The Enlightenment. Friedrich Henle (1809 - 1885) was a German anatomist, physician, and pathologist with numerous anatomical structures presently credited to his name. Similar to other great scientists of the era, including Charles Darwin (1809 - 1882), Henle lived, worked, and contributed to one of the most dynamic periods in scientific history. When Henle first described the ligament (Figure 1) that would eventually carry his name in his 1871 work, Handbuch der Systematischen Anatomie des Menschen, the pelvis was in its fledgling days of being studied [1].

Since 1871, Henle’s ligament has been confused with many other local structures and has even had its entire existence questioned. In this paper, we will review the embryological derivation of Henle’s ligament, history of the terminology associated, and clinical relevance. We will specifically discuss a sickle-shaped bundle of fibers on the lateral border of the rectus sheath formed from the fusion of the internal abdominal oblique’s aponeurosis and the transversus abdominis aponeurosis, which contributes to the posterior wall of Hesselbach’s triangle. As we will discuss, much of the confusion in terminology appears to stem from a desire to label a structure that has relatively drastic variability among individuals.

## Review

Search methods

A search for literature relevant to the structures near the superficial part of the inguinal canal was conducted from July 23 to July 26, 2018 using PubMed, Google Books, and Google Scholar. Queries included, but were not limited to, “Henle’s ligament,” “Jacob Henle 1871,” “falx inguinalis,” “conjoined tendon,” “conjoined area,” “conjoint tendon,” and “conjoint area.” All literature was evaluated in English.

A historical perspective

Ironically, Henle was an anatomist famously against eponymously named structures [2]. In his work, Henle’s ligament was referred to as “ligamentum inguinale internum mediale” [1, 3]. Henle’s precise naming of this structure dispels confusion regarding its location. Scholars of the inguinal region began further investigating the anatomy and their discoveries complicated what was previously known as true—the location, attachment, and even the existence of this ligament.

After Henle, a significant advancement in the understanding of the regional anatomy was carried out by Braune [4]. Braune’s analysis of Henle’s drawings found an incomplete dissection from Hesselbach’s. He more distinctly dissected the posterior aspect of the anterior abdominal wall of the abdomen and described the aponeurosis falciformis (m. recti abdominis) and ligamentum interfoveolare, the latter of which is commonly known as Hesselbach’s ligament [4]. The 1917 text, “Anatomical Names, Especially the Basle Nomina Anatomica,” further proposes the renaming of aponeurosis falciformis to falx inguinalis. This differentiation of fibers from the same aponeurosis and the addition of terminology will repeat itself throughout history, leading to the current confusion and misuse of terminology [5].

In the same period, a 1919 anatomy atlas by Toldt used the terms falx inguinalis and Henle’s ligament synonymously, clearly demonstrating the structure along the lateral border of the rectus aponeurosis as it inserts onto the pubic bone [6]. There is no visible connection lateral to the pubic bone along the inguinal ligament as Henle’s ligament is presently defined.

By 1938, McVay and Anson labeled the falx inguinalis as an artifact of dissection. Their argument was that unless the inguinal ligament was retracted and the inferior portion dissected, the falx inguinalis would not be apparent as previous authors had described because it did not have its characteristic falciform shape unless tension was applied to fibers contributing to the cremaster [7].

Curiously, in 1940, McVay and Anson published a piece with multiple plates in which they labeled the falx inguinalis, again keeping it synonymous with Henle’s ligament [8]. They did not directly discuss their reasoning for no longer calling it an artifact but described it as so thin it was only visible by transillumination. It should be noted that McVay and Anson’s 1940 text also states that by this point in time, the terms inguinal falx and conjoint tendon are used synonymously by British and American authors [8].

According to Skandalakis et al. in 1989, “buried among the names of structures real or imaginary in the inguinal region is the term conjoined tendon,” clearly demonstrating the complexity and variation of the region [9]. In this publication, they described a similar history of the terms falx inguinalis and conjoined tendon being used synonymously since the early 1900s. As Skadalakis et al. described, the incorrect terminology in the region tended to be strongly associated with surgical training [9]. The rectus abdominis aponeurosis had been widely used throughout the late 20th century for hernia repairs before inserting a mesh became the industry standard. Because of the common nature of this procedure, and because the lateral portion of the aponeurosis was sutured to tighten the superficial inguinal ring, surgeons expected to see the conjoined tendon and, therefore, documented its presence. In fact, according to Nyhus and Hollinshead, the conjoint tendon is found in only 3-5% of specimens [10-11].

Accordingly, in 1989, Skandalakis et al. appeared to propose a much clearer, though less specific terminology: “conjoined area” [9]. As the falx inguinalis was often misnamed for the rarely present conjoint tendon, this may be the best terminology for the region. Finally, these authors determined it was best to describe the rectus sheath’s lateral expansion using the term “Henle’s ligament” [9]. As they claim, the term was originally used synonymously by anatomists and clinicians with falx inguinalis, but given the current synonymous usage of falx inguinalis and conjoined tendon, naming it Henle’s ligament is the clearest terminology.

In 1989, Skadalakis et al. made it clear that the terminology of the inguinal region has long been controversial, given the anatomical variation of attachments in this area. Additionally, given the relatively insignificant clinical applications of differentiating the conjoined tendon, falx inguinalis, and Henle’s ligament, they strongly affirmed calling the lateral portion of the rectus sheath the conjoined area [9].

The 2000 text, "Surgical Anatomy and Technique: A Pocket Manual" by Skandalakis et al. no longer made mention of the conjoined tendon [12]. In fact, the authors only discussed a conjoined area and, contrary to their 1989 publication, established that the terms falx inguinalis and Henle’s ligament be used synonymously again [12].

In 2011, a group of Australian researchers published a paper attempting to bring clarity to the differentiation of local structures of the inguinal region [13]. Unfortunately, they began using archaic terminology by once again using the terms "conjoint tendon" and "falx inguinalis" synonymously, going so far as to label an illustration with both terms directed at the same structure. By doing this and not mentioning the term conjoined area, they disrupted Skandalakis et al.’s repeated attempts since 1989 at making terminology of this region more succinct [9, 12].

This regression in use of terminology is even more peculiar given the efforts to create cogency within the terminology by the highly regarded 2004 textbook "Surgical Anatomy: The Embryologic and Anatomic Basis of Modern Surgery" [14]. Similar to their 1989 publication, Skadalakis et al. clearly defined and differentiated the conjoined tendon, conjoined area, and ligament of Henle, which they now use synonymously with falx inguinalis [9].

Finally, the fourth and most recent edition of "Surgical Anatomy and Technique" published in 2013 reaffirms the original 1989 Skandalakis et al. proposition of describing the region as the conjoined area and follows their 1999 postulation that the terms "Henle’s ligament" and "falx inguinalis" be used synonymously to describe the insertion at the pecten pubis of the lateral and vertical extensions of the rectus sheath [15].

Finally, we must make a note of how the 41st edition of "Gray’s Anatomy" and 6th edition of "Netter’s Atlas of Human Anatomy" illustrate this regional anatomy. "Gray’s Anatomy" makes no mention of the falx inguinalis, Henle’s ligament, or the conjoined area [16]. Rather, the text only describes the conjoint tendon and has an illustration showing it on the lateral border of the rectus sheath. Unfortunately, "Netter’s Atlas of Anatomy" also makes has no illustration of Henle’s ligament. Similar to "Gray’s Anatomy," it only shows the conjoint tendon and uses the term inguinal falx synonymously [17].

Embryology

The development of the rectus sheath begins with the establishment of a somatopleure along the ventral wall. Following this, around the fifth to sixth week of development, myotomes begin infiltrating the somatopleure and establish dorsal and ventral walls of the thoracoabdominal wall; these are then termed the epimerion and hypomerion, respectively [18]. The external abdominal oblique, internal abdominal oblique, and transversus abdominis muscles arise from the lateral portion of the hypomerion as do their aponeuroses [18]. Therefore, Henle’s ligament, as defined as the lateral portion of aponeurosis formed by the internal abdominal oblique and transversus abdominis, is a derivative of the hypomerion [19].

Embryology of the region has also been used recently to try and elucidate the presence of Henle’s ligament. A study analyzed 10 male and 10 female Korean fetuses at 14 - 20 weeks gestation to determine the presence of Henle’s ligament through development [3]. Their findings showed complete absence in all of the male fetuses, with Henle’s ligament appearing in six of their 10 fetuses [3]. This closely matched the previously described 30 - 50% frequency described by Skandalakis et al., especially given the 2013 study’s small sample size [3, 15]. The fetal study also acknowledged that female fetal development of the inguinal and gonadal region occurs much more rapidly than that in a male fetus and may contribute to the variation seen in their results [20]. Relating to this study, it should also be noted that variation in regional anatomy between the U.S. and Chinese populations has been found [21].

Adult structure

It is not debated that Henle’s ligament is found on the lateral border of the rectus sheath’s aponeurosis formed by the internal abdominal oblique and transversus abdominis muscles. The literature is unclear, however, on the attachment site of this aponeurosis. As stated, Skandalakis et al. described the insertion at the pecten pubis [12]. LeBlanc et al. define Henle’s ligament as inserting at the iliopubic tract [22]. These authors also used the terms conjoint tendon and falx inguinalis synonymously to describe the insertion of the lateral portion of the rectus abdominis on the pubis [22].

LeBlanc et al. provided statistics with illustrations on the variation found in the insertion of the lateral rectus sheath [22]. Unfortunately, the illustrations do not show or differentiate the insertion point between the pubis and the iliopubic tract; given this is how the authors differentiate what they term the conjoint tendon/falx inguinalis and Henle’s ligament, it is challenging to glean statistics from the illustrations. The text also describes an increased prevalence of the conjoint tendon relative to other studies. It does not acknowledge a differentiation between the conjoint tendon and conjoined area; this may decrease the validity of their statistics.

Other major texts simply acknowledge the discrepancies of terminology in the literature and push toward a common clinical directive as related to the lateral border of the rectus sheath [23].

Clinical relevance and imaging

Historically, the clinical relevance of structures in the conjoined area relates to direct inguinal hernia repair. Through the late 1990s, the primary procedures used to repair direct inguinal hernias were open and included McVay, Bassini, and Shouldice techniques [24]. Each of these involved suturing through the conjoined area to repair the weak floor of the inguinal canal [25]. These once commonplace techniques were relatively abandoned after better outcomes using a mesh were demonstrated in the early 2000s [26]. This correlates with Skandalakis et al.’s 1989 assertion that misnamed structures of the region, i.e., conjoined tendon versus falx inguinalis versus Henle’s ligament, were commonplace, and surgeons believed they should be seeing these structures and suturing through them in cases of direct inguinal hernia repair [9].

Sports hernias

As a part of Hesselbach’s triangle, the conjoined area physiologically plays a role in the support of abdominal contents when there are increased intraabdominal stresses. The proposed mechanism is described as a “shutter” effect where the aponeurosis of the transversus abdominis suddenly tenses when there is increased abdominal pressure [27]. Therefore, the current clinical relevance of the conjoined area is in weightlifters and athletes who play sports involving excessive hip rotation, sudden abdominal tensing, and significant stresses on the hip joint [28]. One of the places these pressures especially add stress is the anterior abdominal wall, affecting the conjoined area [29].

Although the diagnosis of a sports hernia is largely clinical, imaging may be sometimes useful. The literature describes the conjoint area as being best visualized using a plain x-ray with the patient in the flamingo position or with ultrasound. Additionally, CT and MRI may be used, though they are considered inferior to ultrasound [30-32].

Regarding treatment after prevention through screening (by testing hip mobility), the first line of treatment for a sports hernia is conservative treatment. This entails resting the joint for a minimum of four weeks if the athlete is in season, corticosteroid injections, and injections of platelet-rich plasma. Additionally, work with a physiotherapist for three to six months is advised. If conservative treatment does not alleviate symptoms of groin pain, surgery is recommended. Similar to the repair of an inguinal hernia, a mesh is sutured to the area to provide additional support [30].

## Conclusions

As we have seen, the terminology associated with the lateral wall of the rectus sheath has a complicated history. The terms conjoint (also spelled conjoined) tendon, falx inguinalis, and Henle’s ligament refer to different structures according to the author’s background as a clinician or an anatomist. We have demonstrated that from an anatomist’s perspective, the conjoint tendon and falx inguinalis are consistently demonstrated as the same structure on the lateral portion of the rectus sheath. On the other hand, those writing literature directed at clinicians avoid the use of the term conjoint tendon as it is a rarely occurring structure and use the term ligament of Henle and falx inguinalis synonymously. Our findings indicate an appropriate term for the lateral portion of the rectus sheath is the conjoined area. Additionally, the terms “ligament of Henle,” and “conjoint tendon” have clear definitions when those structures are present. However, the term "falx inguinalis" being used synonymously with the ligament of Henle and the conjoint tendon is not appropriate and we should consider making this term obsolete.
